# Bibliographical Notices, &c.

**Published:** 1847-01-01

**Authors:** 


### Bibliographical Notices.

I. Observations on Hydropathy, with an Account of the Principal Cold Water Establishments of Germany. By *J. Stevenson Bushnan*, M.D. 12mo. pp. 170. Churchill, 1846.

II. A Review of Homœopathy, Allopathy, and Young Physic. By *L. M. Lawson*, M.D. Professor of General and Pathological Anatomy and Physiology in Transylvania University. 8vo. pp. 33. Lexington U.S., 1846.

In the first of these works we have another example of a regularly-educated physician coming forward as a warm advocate of hydropathy. As, however, he resides in that town of baths, and general resort of dissipated idlers, Wiesbaden, he is more excusable than are others nearer home. If the dedication of his work to Sir James McGregor, with the ardent hope that hydropathy will be introduced into our military hospitals, is authorised, we are indeed surprized and grieved: we prefer, however, until better informed, to regard it as a piece of gratuitous impertinence. In the work we find nothing that has not already been stated upon the subject, the author indeed, having himself had very little experience in this mode of treating disease, seems to have collected his materials chiefly from the gossip of those he met with, and books already published.

The disposition to meet impostors half-way, and thus minister to the delusions of the public, whether manifested by medical writers or medical reviewers, is a painful subject of contemplation for the well-wisher of his profession. A degree of credulity in the reception of statements is sometimes exhibited by medical men, which certainly the better-informed of their patients are surprized at witnessing; and assuredly never at any period of our critical career have we felt so forcibly the urgent necessity of fortifying the minds of our students by careful preliminary discipline, so as to render them, when they enter their career of life, better able to detect the fallacies which will beset them, and discern the path which professional honor and the interests of mankind require them to follow. Because a number of chronic cases, frequently if not generally the consequences of idleness and debauchery, have benefited by the employment of cold water, and the accompanying (frequently the truly curative ones) conditions of its application, the means is forthwith invested with all the dignity of a general method of cure, and received with more or less favour, however empirically recommended, by certain members of our profession. These gentlemen endeavour to salve their consciences by the statement, that hydropathy must be employed as one of the instruments of medicine and by legitimate practitioners, adding that, cold water has always been a powerful agent in the hands of medical practitioners. It has so, and although Currie's reports of its efficacy by their exaggeration led much to its desuetude, yet it probably may be still more extensively employed with advantage. But does this furnish a reason or excuse for writing books or articles communing with the public upon the matter; or any pretext for sanctioning an extent of application implied by the erection of special establishments? Is it not certain that in such, many must be the cases treated with positive detriment to their well-doing, whether their treatment be conducted by ignorant quacks, or by ci-devant practitioners converted into mere specialists? Will not the spirit of system induce many a rash experiment to be tried which ought never to have been ventured on, and is not the list of such already alarmingly large? We maintain that, in the presence of the prevalence of these systems of quackery, rigid abstinence from all means which may tend to their propagation is the bounden duty of every medical man: knowing, as he does, that however qualified his sanction might be, it would be received without its exceptions, and rendered the means of ensnaring numbers in proportion to the height of his reputation and the extent of his influence. The present co-partnership of regular practitioners, charlatans, amateur doctors and auto-doctors is mischievous and derogatory in the extreme.

Of Professor Lawson's *brochure* we can speak in the highest terms. It is a searching and unanswerable exposure of the fallacies contained in Dr. Forbes' pamphlet, commented upon in our last number. As several anonymous approvals from the United States of the doctrines advanced had been published, we are pleased to find so able a refutation from the pen of a well-known professor of that country. He adopts precisely the same line of argument we entered upon in the article alluded to, but with a degree of extension and illustration our space did not admit of. We have only room for one extract, but that is an important one.

" Dr. Forbes surely could not have perceived the full force and bearing of his opinions, nor the exact import of the denunciations of the regular practice; otherwise, we are constrained to believe, his language and sentiments would have been more guarded, and less calculated to give offence to the profession and support to empiricism. We speak decidedly upon this subject, for we feel much: and whatever antithetical opinions we may express, must be ascribed to a strong sense of injustice, rendered peculiarly forcible by surrounding circumstances.

" The United States, however much we may admire its institutions and economy, must be regarded as the very elysium of quackery. Here, unrestrained, they assume an equality, and in point of law possess it, with the most enlightened and scientific; and by fraud and deception too frequently triumph and grow rich, where wiser and better men scarcely escape starvation. It is no uncommon event to witness an outlandish homœopath rivalling whole communities of the most enlightened and worthy practitioners; and this does not result from any defect of the common system, but depends more immediately on the gullibility of the world in general, and of our communities in particular. These practitioners, cunning and ever on the alert, have already seized on Dr. Forbes' concessions in favour of their success, and with triumphant jeers throw them into the very teeth of the regular school. The *steam-doctor*, and other grades of *botanical* practitioners (a class unknown in England) come in for a large share of glory, and these men, too, lug in Dr. Forbes's opinions to sustain their limber-jack, ricketty concern. The following language, extracted from an article just put forth, under the auspices of the Botanical Association, may serve to shew the state of things.

' It has been clearly proved and admitted by the Editor of the British and Foreign Medical Review, that homœopathy has been quite as successful in the treatment of every variety of disease as the orthodox system, and although he does not admit its superior success, there are many practitioners who have tried both systems, and who confidently assert its superiority. If the old system, with all its resources, cannot confessedly accomplish more than a system which is considered entirely negative and void of effect, it is surely time that our Colleges were looking out for reform. As a pioneer in his cause, we hail the new Cincinnati School.'

" It may be said, however, that Dr. Forbes is not answerable for these results: that he not only has the right, but it his imperative duty to speak the truth, regardless of the sect on which he may fall most heavily. This may be; but he has told not only the *truth*, but *much more* than the truth: and it is this superabundance of expression, and those ultra and unguarded opinions, that will work such unfavourable results for the profession. This ultra course will wholly fail to improve the profession, while it will do more to build up and sustain downright quackery, than half a century of labour by those friendly to these false systems. An opponent's favorable testimony is always laid hold of, and exerts unbounded influence."

LIEBIG'S QUESTION TO MULDER, TESTED BY MORALITY AND SCIENCE. By Dr. *G. T. Mulder*, Professor of Chemistry in the University of Utrecht. Translated by Dr. *P. F. H. Fromberg*. Octavo, pp. 122. Blackwood, 1846.

IN another page of our present number we have endeavoured to do justice to what we consider the undoubted merits of the celebrated Professor of Chemistry at Giessen; and much do we regret that so high a reputation as he has achieved should be sullied by an overbearing and capricious conduct, which he is much mistaken in thinking will be tamely submitted to. As Editor of the now much read "Annalen der Chemie und Pharmacie," he makes its pages the vehicle of virulent abuse and unmitigated depreciation of every chemist, however celebrated, who does not bow his head low to the despotism he believes his popularity has enabled him to establish, or dares to express an opinion adverse to the maintenance of his autocracy. And yet the views of this arch-philosopher are sufficiently unsettled upon important subjects of investigation to have taught him a more diffident mode of gainsaying those who differed from him. Every one must recollect what prominence he attached, in his first edition of the Animal Chemistry, to the discovery of Protein by Mulder, the steps of whose analysis he declared he had repeatedly verified. Well: without a word of explanation in note or text, the subject in the present edition is wholly passed over as if it had never been mooted; and in the Annalen for Jan. 1846, he declares that that which he some years since looked upon as a highly important discovery is *non est inventus*, and coolly asks Mulder how he could possibly have ever believed in, or can prove the fact of, its existence. The present pamphlet is intended as an answer to such question. Its being asked at all seems to have arisen from the fact of Mulder having, in his "Physiological Chemistry," ventured to doubt the accuracy of some of the views of his former patron. All became changed, and Liebig says, in a letter to the author, he is content to take his share of the blame for the adoption of the pseudo-discovery. In the same letter (May 1846) Liebig reminds him—

"'If you look back a few years, you will find that I was the first to do justice (!) to your researches on animal substances. Your results had for years (!) been published in Berzelius' work, and no person understood their meaning. I still think myself fortunate that it was I who first turned the attention of chemists and physiologists to them.'

"How am I to understand this? *Liebig even now thinks himself fortunate* that he has turned the attention of chemists and physiologists to results which are so incorrect, (that is to say, *at present*, for before, *they had all been corroborated*,) that most of them appear to be *false*. The letter continued, 'What we want is not supplied by your analyses, of which time will shew that the greater part (*all* was blotted out) are false.' If the latter statement be true, what ground was there for Liebig's happiness, because *he* had fixed the attention of chemists upon these *false* results? Can it be called justice when *false* results are presented to the world as accurate." P. 38.

The question has furnished Mulder, in this his reply, an occasion for presenting a very complete and yet succinct account of the chemical history of Protein, which we recommend to the notice of such of our readers as are interested in the discussion. It is difficult, indeed, to discover the grounds for Liebig's change of opinion in the face of analyses so often repeated by various hands. For our own parts, we have long been convinced of the soundness of the following observation, made by Dr. Prout in the last edition of his Bridgewater Treatise.

"An attempt has recently been made to shew that albumen, fibrin, and casein contain a certain common fundamental proximate element, to which the name of *Protein* has been given. That such a common proximate element may be derived from albumen, fibrin, and casein, is not denied. Viewed, indeed, in connection with organization and life, the supposition that some common proximate element adapted for ulterior changes exists in animal bodies is very probable; and accords well with the simplicity of natural operations. But that a substance obtained like protein by the rude and disorganizing processes of common chemistry, should be that proximate element; or that such a substance should ever be employed at all in vital operations without undergoing the preliminary assimilating processes, is more than we are at present disposed to admit."

This is to dispute the value, not the reality, of the substance obtained by Mulder. Liebig attributed formerly an exaggerated importance to that which he now declares he is unable to obtain!

---

Chemistry of the Four Seasons. An Essay principally concerning Natural Phenomena, admitting of Interpretation by Chemical Science, and illustrating Passages of Scripture. By *Thomas Griffiths.* Octavo, pp. 490. Churchill, 1846.

Stimulated by the success which attended the publication of some lectures upon the "Chemistry of the Four Ancient Elements," which he had delivered before the Queen, the author, wishing to strike while the iron is hot, has brought out another work having the illustration of the Four Seasons for its object, also founded upon other lectures delivered to more miscellaneous and less august assemblages. We fear it is but a piece of book-making little likely to profit writer or reader. For those already acquainted with the mere elements of chemistry, it contains little or nothing new; and for those who are uninformed upon the subject, and for whom it professes to be intended, it is too defective in the explanation and connexion of its illustrations to be even intelligible. An abundance of experiments are detailed, and with simplicity enough, and we can easily believe in their exciting the admiring plaudits of the popular lecture-room: but the empty nature of any such exhibitions uncombined with detailed and methodical study of the subject is at once seen when publication of the lectures affords an opportunity of observing the absence of numerous connecting links. The book is utterly devoid of arrangement, so that the retention of the facts it deals with would be no easy matter for the novice. It is true they profess to be grouped according to their relations to the various seasons of the year; but the plan is necessarily very imperfectly carried out; and much that is stated under the head of Spring might just as advantageously be placed under that of Winter or Summer, and *vice versâ.* The only work we are acquainted with for *teaching* the elements of chemistry, that deserves the name, is Mrs. Marcet's Conversations, which indeed well merits the popularity it has obtained.

We cannot, moreover, compliment our author upon his repeated introduction of quotations from Scripture, frequently, too, bearing the merest, if any, reference to his subject. We are willing to believe that he is a sincere admirer of the marvels of the Creation, for a poor chemist must he be who is not, but the continual expression of wonderment, and the terming every natural phenomenon "miraculous," do not convey the impression to the reader he doubtless intended they should.

---

THE USE OF THE BODY IN RELATION TO THE MIND. By *George Moore*, M.D., &c. Small 8vo, pp. 431. Longman & Co.: London, 1846.

Two years ago Dr. Moore published a work, entitled, "The Power of the Soul over the Body," which was so well received by the public as to have reached a third edition. Doubtless he has been thereby encouraged to try his fortune once more upon a kindred subject. Whether he will succeed as well this time, seems to us very problematical. His object, indeed, is a good and creditable one; to shew that the study of physiological science ought to be but one of the avenues to that shrine where alone is to be found that highest and best wisdom, which teaches us our duties not only to ourselves and to others, but also to Him, in whom "we live, and move, and have our being." But many of the topics, selected by the author for exposition or illustration, are so mystical in character, and withal there is such an utter absence of the *lucidus ordo* in their handling and arrangement, that we feel ourselves utterly unable to give any correct idea of the contents of his present work, except by selecting a few passages from different chapters, and leaving the reader to form his own judgment. Here is a specimen of Dr. Moore's physiology.

"The first visible germ of the human body is an opaque spot $\frac{1}{300}$ of an inch in diameter, within the germinal vesicle or egg, which is $\frac{1}{60}$ of an inch in diameter. This germ is the commencement of the whole body. Several corpuscles of the mother's blood are acted on at the same time, and caused to arrange themselves, or their elements, so as to form a new being. There is something in this germ which attracts to itself the materials of which all parts of the mature animal is formed. The germ, then, must contain the power which causes growth, the force which ultimately constitutes the power of the whole body. The development of form is but the manifestation of an inherent power, which, under favourable circumstances, produced by the same might, works out the idea of God in the plan of each creature. Thus the human germ cannot be developed into anything but a human body. It is the microscopic concentration of forces, which, under suitable conditions provided by the creative Mind, becomes the full-grown being. In its first beginning, it is but as an atom of dust moved by the breath of God; in the end, the residence of a distinct spirit, capable of enjoying the attributes of the Infinite. These are facts, not opinions." P. 9.

Dr. Moore appears to hold strange, but certainly very comfortable, notions respecting the absence of physical suffering in certain of the lower tribes of animals.

"However our reason and experience may incline us to think of specific organizations, our reflections on instinct would lead us to a very consolatory conclusion, because it indicates the incessant and boundless benevolence of God. All creatures purely instinctive, such as insects, appear to me to be incapable of positive pain, but abundantly endowed with the capacity of pleasure. Their every action results from direct impression, so as always to be accompanied by a feeling of enjoyment, or a sense of doing what is desired, the desire, the action, and the exciting cause of the action, being connected without interval, and without comparison. Thus an insect, although cut in two, will seize its food with avidity." P. 62.

Dr. Moore is a half-and-half believer in Mesmerism, which, he declares, "is not a whit more puzzling than many common things in Natural History." Subsequently he remarks, that "the inductive experiments of Baron Von Reichenbach* leave us now but little room to doubt than Animal Magnetism is a natural

* For an account of these experiments, see the *Medico-Chirurgical Review* for July of last year.

truth of great value and importance to society, notwithstanding the egregious extravagancies of fancy which have disfigured its history as a science."

The following passage, which contains, nevertheless, some food for not unprofitable reflection, inevitably reminds the reader of an early chapter in the history of Tristram Shandy.

"Our education also, may be said to begin with our forefathers. The child of the morally instructed is most capable of instruction; and intellectual excellence is generally the result of ages of mental cultivation; but degeneracy is most marked at both extremities of society, the highest and lowest classes are those worst educated, both morally and physically speaking. It appears from the examination of juvenile delinquents at Parkhurst by Mr. Kay Shuttleworth, that the majority were found deficient in physical organization, and this no doubt was traceable to the parent stock. S. T. Coleridge said that the history of a man for the nine months preceding his birth would probably be far more interesting, and contain events of greater moment than all that follow it. Southey fancied Coleridge was not in earnest in uttering this startling sentence, but perhaps the words convey too profound a truth for the doctor's former vision. Their meaning will shine out if we reflect on the influence which the mother's and even the father's habits exert on the constitution moulded *in utero.* There the ground-work of all history is laid in embryo, and the seeds of evil there begin to take root, and to vegetate in a genial soil long before they open their leaves to the sky. The soil, indeed, alters not the nature of the seed, but vast is its effect on development, and no one can doubt that the state of the parent determines, in a large measure, the predisposition of the offspring, for predisposition, in fact, signifies only bodily aptitude." P. 97.

There is a curious metaphysico-physiological chapter on "Light in relation to Life." Here is an extract from it.

"We possess proof of the astounding fact, that solar light causes a regular succession of movements in the medium through which it passes, to the amount of five hundred millions of millions in a second; and it is because this vibration acts upon something in our brain capable of vibrating in a corresponding ratio, that our souls are put in such relation to light that we can enjoy vision. The time of different colours, however, is not the same; our sense of sight is affected by red 458 millions of millions of times in a second; by violet, 727 millions of times; and by yellow, 542 millions of millions of times in a second. Of course, therefore, different colours differently affect our souls. Throughout nature, these undulations of light are so modified as to be productive of a vast variety of enjoyments to various creatures, and to operate in such a manner upon their nerves and faculties as to guide them to the fulfilment of those desires which colour and shape contribute to excite." P. 170.

The following remarks on the newest charlatanery of the day—practised although it be, we regret to say, by several members of the profession, and even smiled on by one of our cotemporaries in medical journalism—are judicious, and will recommend themselves to the good sense of the reader.

"The benefit of habitually abstaining from *artificial* stimulants can scarcely be better expressed than it has been by some sudden converts to a simple regimen in the name of hydropathy. From their rapturous language, describing their delights in the feelings of a new kind of life and vigour, one might suppose them to have just escaped the misery of a depraved existence, and to have found themselves, unexpectedly, in some poetic paradise. But there may be intemperance even in the use of water. The ecstasies of hydropathic converts, however, is due as much to excess of enthusiasm as to excess of drinking. Active exercise in fresh air, and a free use of cold water, constitute a plan which every savage, unbewildered by quackish mysteries, knows to be wisest, discreetest, best for securing the blessings of bodily health. But let moderation be known in all things, and despise not the wisdom of Solomon, who tells us that wine has its uses, and strong drink is more suitable than cold slops and wet sheets for a man with a flagging pulse and a sinking heart. A deluge not only renovates, but also destroys; and the Maker of man never designed him to be amphibious, nor to keep his functions in forcible action, like a water-mill under a constant stream, but to enjoy life under a wise use of all that is good, since obedience to Divine law allows of no extremes; and temperance implies *in mediis tutissimus*—an equal danger both from abstinence and excess." P. 229.

---

THE POTATOE PLANT, ITS USES AND PROPERTIES; TOGETHER WITH THE CAUSE OF THE PRESENT MALADY. THE EXTENSION OF THAT DISEASE TO OTHER PLANTS, THE QUESTION OF FAMINE ARISING THEREFROM, AND THE BEST MEANS OF AVERTING THAT CALAMITY. By *Alfred Smee*, F.R.S. Octavo, pp. 170. Ten Lithographs. Longman 1846.

The dreadful visitations termed Famines, arising from the deficiency of some staple article of food, have been of too frequent occurrence in the past history of Europe, and happen still too often in India, to allow of surprize being mingled with the commiseration excited. Vicissitudes of season, negligence of culture, the ravages of insects, or the devastations produced by warfare, have, in the different cases, afforded obvious enough explanation of the calamity. But the destruction of the Potatoe, has not only proceeded with a fearful rapidity and universality, but it has been accomplished by causes which have eluded observation, and which, so far from yielding to the various precautions that have been suggested, seem now to be operating with increased intensity and over a wider sphere. Appearing first at Courtraî at the end of June 1845, the disease rapidly spread over the rest of Belgium and thence into and throughout France and Germany. The counties opposite the mouth of the Thames were those which first suffered from its ravages in Britain, but others soon became involved, and by September and October, it had extended through Scotland and Ireland. The loss of the crop, vast in 1845, became doubled in 1846, and the prospects of the next year are still more gloomy, and even the extirmination of this valuable tuber is looked upon by some alarmists as impending. In such a state of things it is not surprising that the attention of the chemist, the naturalist, the agriculturist, and the political economist in every country of Europe should be directed to the investigation of the etiology of this singular malady, and the means of averting its ravages. Mr. Smee, however, believes that the *surgeon* is the person more especially likely to elucidate the mystery.

"The investigation into the nature of an universal disease among organic bodies belong especially to the practical surgeon. He is investigating disease in every hour of the day, and every day of his life. He is accustomed to weigh the various difficulties which arise in the investigation of a complex organic body; and on that account he is peculiarly suited for the discovery of the cause of an universal malady. The disease in the plant is a death of the vegetable tissue, and the question of life and death especially pertains to the business of a surgeon.

The business of a surgeon is essentially locomotive, and his duties are practised over an extensive space. It frequently happens that I have to traverse London in two or even more directions in a single day, which circumstance has given me abundant opportunities of making my observations in different localities. Moreover, during the summer months, I was living at Springfield, Upper Clapton, where I had the advantage of a large garden, wherein were several plots of potatoes, which I was in the habit of observing the first thing in the morning, again on my return from London, and frequently the last thing at night. In the neighbourhood, moreover, were larger potatoe grounds, where I used to enjoy the air, and study the disease in the evening: and it has curiously happened, that I have made my observations on the potatoe plant in the same garden in which I conducted the experiments for my former work on 'Electro-Metallurgy.'" P. 11.

We must confess that this statement (which we fear some of our country readers will think savours somewhat of Cockney-land) does not at all convince us that this subject of investigation falls legitimately within the sphere of the surgeon or physician. When we consider that the man engaged in active practice is even incompetent to minute investigation of the causes and nature of the diseases of the human frame, and is chiefly occupied in following out indications furnished him by the laborious inquirers who devote themselves to such pursuits, we can easily see his utter unfitness for pursuing a train of inquiry which demands complete information in several branches of knowledge which he could only have acquired at the expense of others of a more practically important character. However this may be, we cannot congratulate Mr. Smee upon having discovered any efficient cause of this terrible malady. He believes this to be induced by a species of *Aphis,* "which punctures the leaf, sucks the sap, and destroys the relation between the leaf and the root, thus causing the leaf or some other part of the plant to become gangrenous, or in other words to die." He states that, in the potatoe grounds alluded to above, he observed multitudes of this aphis, which, from its presumed ravaging powers, he terms *Vastator.* The conferring a new name for hypothetical reasons upon a species, which he states to be identical with the *Aphis Rapæ* of Curtis is quite unjustifiable, and must be protested against, for if such a practice were generally followed, endless confusion in the study of Natural History would result. When we consider the multitudes of blasted potatoe plants which have been examined in all stages of the disease by competent observers without their presenting any traces of this insect, as also its *comparatively* innocuous effects upon the various other vegetable it infests, it at once becomes evident that Mr. Smee has fallen into the too common error of mistaking a coincidence, or perhaps even an effect, for a cause, and consequently has thrown no useful light upon this obscure subject. His work, however, contains a good account of the Potatoe as an article of diet, which has been compiled with great care and will be read with interest and advantage.

---

NOTES ON THE EPIDEMIC CHOLERA. By *R. Hartly Kennedy,* M.D. &c., late Physician-General and President of the Medical Board, Bombay. Second Edition, revised. Small 8vo. pp. 279. London: Smith, Elder and Co. 1846.

THIS is a mere reprint of a work that was published upwards of twenty years ago. Dr. Kennedy tells us that, being retired from professional life, he can have no personal object to gain by re-appearing before the public, save the wish to be of service to the profession generally, by making them acquainted with his views on the pestilence of India which seems to threaten a re-invasion of Europe. This can be the only plea (if indeed it is one) for the utter omission from its pages of all new matter. There is not so much as a passing allusion to what has been said or written on the subject, since the date mentioned; so entirely enamoured, we suppose, is Dr. Kennedy with his own views and opinions.

Dr. K's. theory of Cholera may be gathered from the following passage:—

"I consider a nervous derangement, similar to concussion of the brain, to be the disease, how induced I know not, following the above inexplicable shock sustained by the constitution; and the collapse and spasms to be symptomatic of the disorder of the brain; and finally, I consider the purging and vomiting to be no part of the disease, but the struggle and effort of Nature to relieve the constitution, and cast off the noxious principle which is destroying it. For the treatment of such a disease, the indication is distinctly apparent to relieve the brain by bleeding, and to induce the sanitary process of vomiting and purging where they do not exist, or to moderate them when violent." P. 57.

He considers Cholera to be quite as infectious as Small-pox or the Plague!

"However aware I am of the delicacy of the ground on which I have advanced, and however strongly sensible that nothing would give me more sincere happiness than to be able to change the opinion I entertain, I am under the necessity of ending this part of the discussion with observing, that to the best of my judgment, I know no character belonging to any contagious disease which Cholera does not appear to me to possess; and that, if it be not contagious, I know no other disease which I should be inclined to consider so." P. 75.

Dr. K. regarding the vomiting and purging (more especially the former) to be, primarily, only sanitary efforts of Nature to relieve herself from the prostrating enemy that she has to contend with, wisely cautions his readers not to seek to check these evacuations at once by powerful astringents and sedatives. He is favourable to the use of Emetics, and also of mild Purgatives in combination with opium and other means; bleeding having been premised in the beginning of the attack. His favourite formula was the following:—

"I mingled of castor-oil and honey each six ounces; tincture of opium, twelve drachms; and camphor mixture, ten ounces and a half. For the pills, I powdered three ounces of camphor by friction, adding the necessary small quantity of spirit of nitric ether in lieu of common alcohol; then dissolving two ounces of gum opium in tenacious mucilage of acacia gum, I had the whole effectually beaten into a mass, and divided into 480 pills, each of course containing three grains of camphor and two of opium.

"The directions were, that the patients, as soon as they were attacked, should be made to drink plentifully of hot water, which being vomited up, and the irritation of stomach for a moment relieved, two ounces of the mixture were to be administered; if sickness returned, a copious draught, or at least a pint, of warm infusion of the toolsee (Ocimum gratissimum), an herb always at hand as the sacred plant of the Hindoos, should be given. This infusion was generally at first vomited up; but the second draught, if it did not allay the sickness, always came up with less of pain and spasms. One of the pills was then to be given, and, if vomited up, to be repeated after each vomit. When the stomach was soothed by the pills, and, one ghurry after the last vomit, a second dose of two ounces of the mixture was to be administered, and the patient was permitted to quench his thirst, or allay the burning heat at the stomach, by drinking equal parts of milk and thick rice-water cold; after which, in ordinary cases, the patient would sleep. If these did not, however, give relief, but the vomiting continued, they were directed to apply scalding water to the scrobiculus cordi, so as to raise an instantaneous blister." P. 232.

---

The Microscopic Anatomy of the Human Body in Health and Disease. Illustrated with numerous Drawings in Colour. By *Arthur Hill Hassall,* Author of a "History of British Freshwater Algæ," &c. Part V. London: Samuel Highley, 1846.

The author has cancelled some of the earlier plates, in which, owing to some mismanagement in striking them off, bad impressions were obtained; more attention has also been paid to the colouring. These are great improvements and have added to the utility of the work. The Part just published (December) contains a very accurate account of the chemical and microscopic qualities of the milk, embracing the observations of Simon, Henlé, Donné, and other authorities; and illustrated by original drawings of human milk and colostrum. The seminal fluid is also described, and two figures are given showing the seminal granules and spermatozoa. When completed, the work will be of much service to those who are commencing the study of microscopy, affording a concise descriptive account and illustrations of the several fluids and tissues of the animal body.

---

The Handbook of Human Anatomy, General, Special, and Topographical. Translated from the original German of Dr. *Alfred Von Behr*, and adapted to the Use of the English Student by *John Birkett*, Fellow of the Royal College of Surgeons of England, and Demonstrator of Anatomy at Guy's Hospital. London: Longman & Co. 1846.

The handbook of Dr. Von Behr belongs to a class of works for which we must confess we have little affection; in which the ingenuity of the author is taxed rather to contrive how he may best meet the desires of idle students, than in advancing the science of anatomy. The author disavows such an object, and we have no reason to doubt his sincerity; but still the general tendency of these meagre manuals is to encourage a superficial taste in a case where full and complete knowledge is pre-eminently demanded. We regret that we should have occasion to make these remarks, because the volume before us contains much valuable matter; and particularly because the plan of the work, embracing general, special, and topographical anatomy, is excellent. Such a work, judiciously executed, would be a boon to the medical student; and this hand-book, if it were somewhat amplified, would have been just the thing that is required at the present moment.

The details relating to structural anatomy, as might be expected in a work emanating from Germany, form the most valuable part of the treatise; and the most defective section is that on the topographical anatomy. Mr. Birkett has, indeed, by his notes, greatly improved this part of the text; and we only regret that he did not furnish a larger portion of information, as there is no branch of anatomical science that has been more successfully cultivated in this country, or is more attractive to the student.

Although there are the drawbacks we have noticed, the hand-book of Dr. Von Behr will assist English students, especially in that department, minute anatomy, which at present is often neglected; and with this conviction we recommend this manual to those for whose use it is more particularly designed.

---

Principles of Human Physiology, with their chief Applications to Pathology, Hygiene, and Forensic Medicine. By *William B. Carpenter*, M.D., F.R.S. &c. Third Edition. London: John Churchill, 1846.

It is satisfactory to find, by the appearance of a third edition of this standard work, that the science of physiology, and its inseparable adjunct, minute anatomy, are now studied in our schools on philosophic principles. Whatever may have been the doubts in some quarters concerning the utility of microscopy, they must have yielded ere this before the accurate and great results that have been secured by the labours of English and Continental observers. Structural anatomy and organic chemistry it is apparent, are the principal means by which further progress is to be attained; and this indeed might be inferred by the history of all other similar branches of inquiry. We have reached a point in human anatomy, at which all that can be studied by the unaided eye, has been well nigh exhausted; so that, unless other, and more subtle, methods of research be devised, we must be content to halt in the path of discovery. If any other proof were required of the position here assumed, it would be amply furnished by the volume before us. Dr. Carpenter, himself a most successful microscopist, has collected together a vast amount of valuable facts, which, combined with chemical analysis and comparative anatomy, constitute the basis on which these philosophic "principles" repose.

As we have on a former occasion (see Med.-Chir. Review, No. II. April 1843) noticed at some length Dr. Carpenter's work, we shall merely now state that by a revision, which bears in every chapter the impress of great research and great judgment; and by the addition of new matter to the amount of at least a hundred pages, the author, without augmenting either the bulk or the price of the volume, has enriched it by the most important of the numerous contributions which have been made to Physiological Science within the last two years. It is not the student alone who requires the aid of a work at once so recondite and lucid as that before us; the practitioner, in those obscure cases, implicating important organs, and so often recurring, will in these pages find the means for forming a correct diagnosis, and consequently, for arriving at a rational mode of treatment. With these convictions, we urgently advise our readers to add to their libraries this last and extended edition of Dr. Carpenter's Principles of Physiology.

---

### Transactions of the Medical and Physical Society of Bombay. For the Year 1844.

This Number contains papers by Messrs. Murray, Hunter, and Impey, and by Drs. Arnott and Morehead, the latter gentleman (as on some former occasions) contributing more than one-half of the whole matter. His situation in the European General Hospital at Bombay, affords him an extensive field for observation, and no zeal is wanting on his part to turn his opportunities to good account. His zeal in prosecuting the morbid anatomy of intertropical diseases is most meritorious.

From his paper on the "Pathology and Treatment of Dysentery," we select the following passage bearing upon the important point as to the connection of this disease with Hepatic Suppuration—a subject that has been repeatedly brought under the attention of our readers in recent numbers of this Journal.

"Complication with abscess in the liver constitutes a great proportion of the fatal cases of chronic dysentery, and in such the dysenteric symptoms are not unfrequently in no respect more urgent than in cases which without this complication do well. They may indeed even improve for a time but they do not so steadily, and the increasing emaciation and languor are greater than the degree of the dysenteric symptoms can satisfactorily explain. If, under these circumstances, we detect an evening febrile accession, there are very good grounds for suspecting that abscess has either formed or is forming in the liver, very shortly to become evident by the usual symptoms of uneasiness of the right side, followed by distinct fulness and tenseness at the margin of the right false ribs. A reference to the series of cases published by me in this No. of the Society's Transactions and elsewhere, will shew very distinctly that ulceration of the large intestines and abscess in the liver are very often found co-existing. In one set of cases, those ranged by me under the head of dysentery, the affection of the bowels was the primary and prominent disease, that of the liver coming on more or less obscurely as a secondary event. In the other set, the symptoms of hepatitis were the primary and most prominent, those of ulceration of the bowels succeeding and being often not very clearly indicated. But to infer from these facts that the diseases bear to each other the relation of cause and effect, though a plausible enough inference from the exclusive consideration of their morbid anatomy, is untenable as a general proposition,—for it would be to generalize from the fatal cases only, and to leave the successful ones out of account; or to express it otherwise, it would, in the data before us and on the assumption, not a correct one, that in every fatal case of dysentery there was abscess in the liver, be to deduce a general law from 18 per cent. though opposed by 82 per cent. Nor is the force of the statement materially lessened by the admission, which may be made, that it is probable enough that, in some of the successful cases of dysentery, symptoms of hepatic inflammation may have occurred and been subdued, and, in the successful cases of hepatitis, dysentery may have threatened and been averted—for I can state very confidently that this, though occasional, has been by no means a frequent occurrence."

The following remarks on the Calomel and Opium treatment of Dysentery deserve notice:

"The treatment of dysentery by large doses of calomel repeated and continued for some time, on the principle that such doses have a sedative action on the inflamed mucous coat, does not I think at present find much acceptance in practice in this part of India; and I believe that it may fairly be assumed that a system of treatment strongly recommended and at one time generally followed, as this has been, would not have fallen into disuse, unless the expectations formed of its efficacy and applicability had led to disappointment. On this principle I would explain the comparative infrequency of the treatment of dysentery by large doses of calomel repeated and continued for some time. My own belief* is that as a general system of treatment it is inappropriate and very often very injurious."

How comes it that our friends in the East are so tardy in their movements? We did not receive this number, published it is stated in the title-page in 1844, until three months ago.

---

A Tabular View of the Physical Signs and Diagnosis of the Diseases of the Lungs, with a Synopsis of the Signs which occur in each Disease. By *James Turnbull*, M.D. Physician to the Liverpool Northern Hospital.

This tabular view affording a *coup-d'œil* of the various auscultatory, &c., phenomena discoverable in health and disease will prove useful to many, practitioners as well as students, in their investigation of thoracic maladies. We should have liked it better if the rational or subjective symptoms of each had been given at the same time, and if a place had been found for diseases of the Heart, as well as for those of the Lungs.

---

* "In the latest works on Materia Medica, the sedative action of large doses of calomel is assumed on the authority of Tropical practitioners. This is not the place to discuss the general question, nor am I prepared to do so, but I may say that I am not convinced that large doses of calomel possess such action independent of the opium with which they may be combined, and I doubt whether the question ought to be considered as one settled."

